# Affinity, value homophily, and opinion dynamics: The coevolution between affinity and opinion

**DOI:** 10.1371/journal.pone.0294757

**Published:** 2023-11-27

**Authors:** Miao He, Xiaoming John Zhang

**Affiliations:** 1 Yanqi Lake Beijing Institute of Mathematical Sciences and Applications, Beijing, China; 2 Institute for Applied Mathematics, Tsinghua University, Beijing, China; AGH University of Krakow: Akademia Gorniczo-Hutnicza im Stanislawa Staszica w Krakowie, POLAND

## Abstract

We propose two analytical relationships between affinity and opinion change. The first one focuses on value homophily, while the second one incorporates affinity in opinion dynamics. Three analytical test models are derived based on these relationships: the value homophily model, the temporal evolution of opinion summation, and the evolution of opinion difference between two individuals. We test these models using data from a previous experiment, and the results demonstrate their validity.

## Introduction

### Affinity and value homophily

Communication is an indispensable part of our lives. The evolution of opinion through communication has received considerable attention in the past few decades [1–3]. However, changes in feelings towards others can be accompanied by communication. The term “attraction” has been used as a measure of mutual liking between two individuals in Byrne’s work, which studied the influential factors on interpersonal impression and liking [4]. Nonetheless, in some other works [1, 5], “attraction” is used to describe the narrowing of opinion distance (or we refer to as “opinion difference”), which can lead to potential confusion. Therefore, an alternative term is warranted to distinguish these two different usages. In this work, we use “affinity” instead of “attraction” to depict the degree of mutual liking between two individuals.

The development of this work is rooted in a basic assumption: affinity and interpersonal association tendency are positively correlated. The proposal of this assumption is drawn from the social exchange theory [6], which suggests that the tendency of social interactions is driven by rewards and costs. Humans are more likely to associate with each other if high affinity exists, and vice versa.

Homophily effect has been observed, analyzed and reviewed by hundreds of publications [7–11]. When people are similar to each other, they are more likely to associate with each other and the communication among them becomes easier. Lazarsfeld et al. classified homophily into two types: status homophily and value homophily. Status homophily denotes the inclination to associate with people who share similarities in social attributes. These attributes include both society-ascribed characteristics (such as race, ethnicity, sex, and age) and developed characteristics (such as religion, occupation, social class, and education). Value homophily, on the other hand, refers to the tendency to associate with others who share similar values, attitudes, and beliefs, regardless of differences in status characteristics [12]. In this work, we focus on value homophily solely.

The effect of value homophily in opinion dynamics has been analyzed by multiple studies. Acemoglu et al. assumed that the likelihood of opinion update of a target individual is proportional to the opinion difference between themselves and the source individual who influences them, with the introduction of an exponential function of the opinion difference [13]. They presented this assumption with simulation experiments. Later, this assumption was employed by Axelrod et al. in their study regarding political polarization [1].

While there is a large body of research on value homophily, fewer studies have focused on the relationship between opinion difference and affinity. Byrne was among the first to validate the hypothesis that a stranger who shares similar views with a subject would be better liked by that subject [14]. Gubanov et al. proposed that similar values function as a kind of constructive similarity, enhancing affinity between individuals [15]. Parallel conclusions can also be found in [4, 16]. While these studies shed light on how opinion difference and affinity are correlated, the analytical relationship between the two and its empirical validation are still underexplored. In this work, we aim to quantify the change of opinion difference and its relationship to the change in affinity.

### Opinion dynamics models

Opinion refers to a subjective view held by an individual towards a certain issue or topic. An opinion score measures the position of the view on a given scale. For example, for a topic like “The government should subsidize public transport in 0… 100 percent.”, different percentages reflect the varied attitudes held by people on the issue of public transportation support. Such percentages are referred to as the opinion score. Opinion dynamics refer to how people’s views change over time through interactions with one another. Opinion dynamics is a multidisciplinary research field that attracts scientists from different domains and has been applied to diverse fields, including social psychology [17], politics [18], physics [19], internet network sciences [20], business management [21], and more.

Classic opinion dynamics models mainly include three categories. Assimilative models [2, 22] assume that assimilative influence is continually exerted during communication, leading to a reduced opinion difference (referred to as a positive shift or attraction effect). Similarity bias models [3, 23] introduce a threshold to distinguish the conditions under which assimilation occurs. Specifically, opinions tend to shift towards each other when and only when the opinion difference between two individuals is smaller than a given threshold. Repulsive models [24, 25] are in the third category, which assume that the influence can be either positive or negative, depending on the difference. When the difference between two individuals is significantly large, their opinions tend to shift away from each other (referred to as a negative shift or repulsion effect). Several studies [26, 27] incorporate static factors such as demographic attributes into the models.

Most previous works have focused on the effect of opinion difference on opinion evolution, but the role of affinity has not been explicitly included in prior research. We believe that affinity influences the impact that opinion difference has on opinion change. The large affinity-opinion dynamic system among individuals consists of several micro relations that collectively shape its evolutionary behaviors. Given the intricate nature of humanity, it is challenging to provide an accurate picture of the whole structure at present. However, to have a better understanding of this complex system, it is necessary to identify and quantify those underlying micro-relations. Opinion dynamics illustrate the evolution of opinion scores for many individuals within a social network. To understand the mechanisms underlying this evolution, it is a common approach to formulate micro-level opinion interaction rules for pairs of individuals.

Following the directions pointed out by Flache et al. [28], which emphasize the need for empirical data to support the models, we first establish the analytical relationships associated with value homophily and opinion dynamics. Subsequently, we test the proposed models on an experimental dataset. This paper has two objectives. Firstly, it aims to develop an analytical relationship between the affinity score and the opinion difference between two interacting individuals and validate it using experimental data. Secondly, it aims to apply this value homophily relationship to model opinion dynamics and assess the model results using the same dataset.

This paper is organized as follows. The Theories section establishes a relationship between affinity and opinion difference, known as value homophily, and incorporates this relationship into opinion dynamics. Three models regarding affinity and opinion evolution are derived. The section of Experimental data description reintroduces and describes the experimental design and the associated dataset from a previous study [29]. The section of Statistical model tests presents the results of statistical tests conducted on the three models. Finally, the Discussion and Conclusion sections delve into discussions, provide conclusions, and offer insights for future research.

## Theories

### Affinity and value homophily

The affinity score is a measure of mutual liking between a target individual and a source individual. It evolves over time as the two individuals interact and learn more about each other. On the other hand, the opinion score measures the position of an individual’s view on a given scale. The opinion also evolves over time as the individual gains more knowledge about the topic and is influenced by others.

We denote *A*_*ji*_(*n*) as the affinity score that target individual *i* assigns to source individual *j* after the *n-th* round of interaction. Similarly, we denote *B*_*i*_(*n*) as the opinion of target individual *i* on a specific topic measured after the *n-th* round. In an anonymous environment where personal information is not exchanged, except for communication solely based on opinion scores, only the value homophily effect is present, and no status homophily is observed.

To capture the relationship between affinity and opinion difference, denoted as Δ*B*_*ji*_(*n*) = *B*_*j*_(*n*) − *B*_*i*_(*n*), we propose an analytical value homophily relationship. It is represented by Eq 1, where parameter *μ* serves as a value homophily coefficient. This coefficient measures the rate of exponential decay or growth of affinity as the opinion difference between the two individuals increases or decreases.

$$\begin{array}{c} A_{ji}\left(n\right)=A_{ji}\left(\Delta B_{ji}=0\right)e^{-\mu |\Delta B_{ji}\left(n\right)|}=A_{ji}\left(\Delta B_{ji}=0\right)e^{-\mu |B_{j}\left(n\right)-B_{i}\left(n\right)|} \end{array}$$ (1)

where (Δ*B*_*ji*_ = 0) denotes the specific round in which the opinion difference between target individual *i* and source individual *j* equals to zero.

The affinity score *A*_*ji*_(*n*) of target individual *i* for source individual *j* is inversely proportional to the exponential of the absolute value of the opinion difference between them. The affinity score reaches its maximum *A*_*ji*_(Δ*B*_*ji*_ = 0) when the opinion difference vanishes. In the absence of influence from other sources, the ratio of affinity scores between any two rounds of interactions can be obtained by normalizing with a maximum constant.

$$\begin{array}{c} \frac{A_{ji}\left(n+1\right)}{A_{ji}\left(n\right)}=\frac{e^{-\mu |\Delta B_{ji}\left(n+1\right)|}}{e^{-\mu |\Delta B_{ji}\left(n\right)|}}=e^{-\mu [|\Delta B_{ji}\left(n+1\right)|-|\Delta B_{ji}\left(n\right)|]} \end{array}$$ (2)

By taking the logarithm of Eq 2, the value homophily relationship can be expressed as Eq 3.

$$\begin{array}{c} ln\left[\frac{A_{ji}\left(n+1\right)}{A_{ji}\left(n\right)}\right]=-\mu [|\Delta B_{ji}\left(n+1\right)|-|\Delta B_{ji}\left(n\right)\left|\right] \end{array}$$ (3)

We refer to Eq 3 as Model 1, which will be subjected to statistical testing using an experimental dataset in the section of Statistical model tests.

### Opinion dynamics of two interactive individuals

Opinion dynamics involving multiple interacting individuals have been extensively studied in numerous publications [30, 31]. Previous models have taken into account the impact of opinion differences between the source and target individuals on the opinion shift of the target. In this article, we introduce the concept of affinity as a multiplicative factor in this effect. Specifically, the updated rate of the opinion score for target individual *i* after the *n-th* round of interaction is linearly proportional to the product of the affinity score of source individual *j*, as evaluated by target individual *i*, and the opinion difference between these two individuals. The target individual adjusts its opinion with its own current opinion serving as an anchor. The proportional factor can be referred to as the opinion change coefficient, which quantifies the rate of opinion shift after each interaction.

$$\begin{array}{c} B_{i}\left(n+1\right)=B_{i}\left(n\right)+\alpha A_{ji}\left(n+\frac{1}{2}\right)\Delta B_{ji}\left(n+\frac{1}{2}\right)=\alpha A_{ji}\left(n+\frac{1}{2}\right)\left[B_{j}\left(n+\frac{1}{2}\right)-B_{i}\left(n+\frac{1}{2}\right)\right] \end{array}$$ (4)

where $n+\frac{1}{2}$ denotes the intermediate status between the *n*-*th* round and the (*n* + 1)-*th* round.

The affinity score plays a crucial role in influencing the target individual’s receptiveness to persuasion from the source individual and in modifying its opinion score. A lower affinity score of the source individual results in less change in the opinion of the target individual, while a higher affinity score of the source individual leads to a greater change in the opinion of the target individual.

By exchanging the roles of the source and target individuals and considering the influence of individual *i* on individual *j*, assuming *A*_*ij*_(*n*) = *A*_*ji*_(*n*), we can describe the updated opinion score for individual *j* as follows. Recognizing Δ*B*_*ij*_(*n*) = −Δ*B*_*ji*_(*n*), we have,

$$\begin{array}{c} B_{j}\left(n+1\right)=B_{j}\left(n\right)+\alpha A_{ij}\left(n+\frac{1}{2}\right)\Delta B_{ij}\left(n+\frac{1}{2}\right)=-\alpha A_{ji}\left(n+\frac{1}{2}\right)\Delta B_{ji}\left(n+\frac{1}{2}\right) \end{array}$$ (5)

The summation of Eqs 4 and 5 reveals that the summation of the opinion scores of the two interacting individuals remains constant over time, as demonstrated by Eq 6.

$$\begin{array}{c} B_{i}\left(n+1\right)+B_{j}\left(n+1\right)=B_{i}\left(n\right)+B_{j}\left(n\right) \end{array}$$ (6)

In the section of Statistical model tests, we will test this opinion summation model of two interacting individuals, referred to as Model 2, using experimental data.

By subtracting Eq 4 from Eq 5, we obtain Eq 7.

$$\begin{array}{c} \Delta B_{ji}\left(n+1\right)-\Delta B_{ji}\left(n\right)=-2\alpha A_{ji}\left(n+\frac{1}{2}\right)\Delta B_{ji}\left(n+\frac{1}{2}\right) \end{array}$$ (7)

By rewriting Eq 7 using a central finite difference scheme, we can express the change in opinion difference between the two individuals from *n* to *n* + 1 as,

$$\begin{array}{c} \Delta B_{ji}\left(n+1\right)-\Delta B_{ji}\left(n\right)=-\alpha \left[A_{ji}\left(n+1\right)\Delta B_{ji}\left(n+1\right)+A_{ji}\left(n\right)\Delta B_{ji}\left(n\right)\right] \end{array}$$ (8)

This is the model for the change in opinion difference between two interacting individuals between the *n*-*th* and the (*n* + 1)-*th* round of interaction, referred to as Model 3. It will be subjected to statistical testing using the experimental dataset in the section of Statistical model tests.

#### An illustrative example

An example is presented herein to offer a simulation-based showcase of ideas from the models we introduced earlier.

For illustration purposes, consider the opinion dynamics model with I = 20 individuals. Suppose the individuals’ initial opinion levels follow a uniform distribution *U(1,99)*, and their initial mutual affinity scores are calculated based on Eq 1 with *A*_*ji*_(Δ*B*_*ji*_ = 0) = 99 for all pairs.

**Fig 1:**
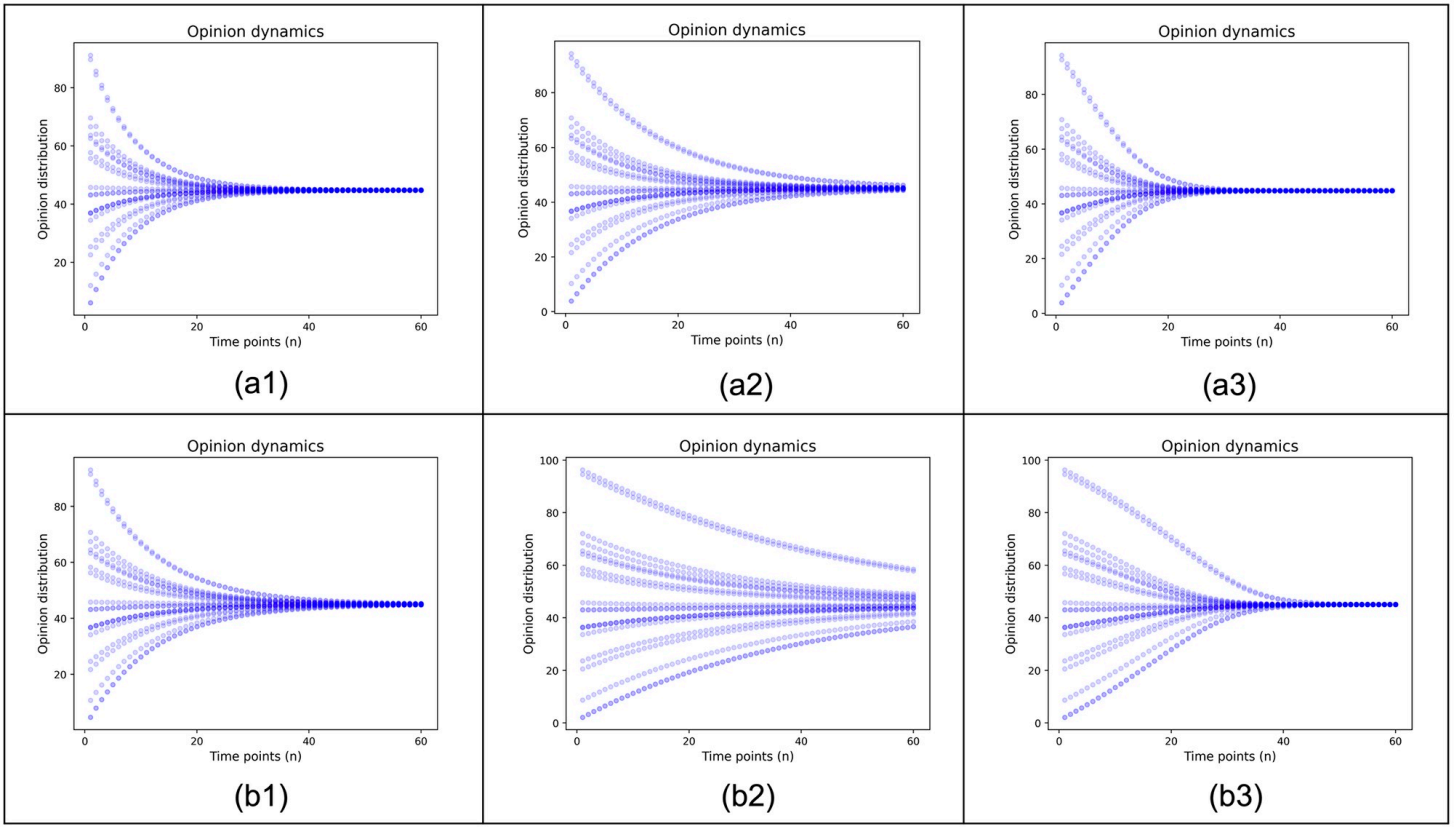
Opinion evolution of 20 individuals. *μ*= 0.02 for (a1), (a2), (a3) and *μ* = 0.04 for (b1), (b2), (b3). (a1) Affinity scores = 59, (b1) Affinity scores = 41 for all pairs and remain the same; (a2) (b2) Affinity scores keep their initial values and remain the same thereafter; (a3) (b3) Affinity scores coevolve with the opinion scores.

Fig 1 shows the evolutions of opinion distributions with two different values of homophily coefficient, and each has three cases: *μ* = 0.02 for (a1), (a2), and (a3) and *μ* = 0.04 for (b1), (b2), and (b3). The simulation was performed for n = 60 time points with the opinion change coefficient *α* = 0.002. After interacting with the others, an individual’s opinion score is updated as Eq 9, which is a generalization of Model 3 from pairwise to groupwise interaction.

$$\begin{array}{c} B_{i}\left(n+1\right)=B_{i}\left(n\right)+\frac{1}{I}\Sigma_{j\in \{1,...,I\}}\alpha A_{ji}\left(n+\frac{1}{2}\right)\Delta B_{ji}\left(n+\frac{1}{2}\right) \end{array}$$ (9)

We use three updating rules for the affinity scores. In (a1) and (b1), the affinity scores of all pairs of individuals are set as the average of the initial affinity scores computed from Eq 1 throughout the simulation. This updating rule corresponds to the classic models where the opinion change coefficient is the same for all pairs and does not change with time. In (a2) and (b2), the affinity scores of all pairs are kept unchanged. This rule allows each pair to have its own affinity score, which does not change with time. In (a3) and (b3), the affinity score of each pair is updated every time point according to Model 1, as the opinion difference of the pair changes. This rule allows the coevolution of affinity and opinion scores.

Fig 1 illustrates the following points,

- The opinions of individuals with higher affinity scores 59 in (a1) converge faster than those of individuals with lower affinity scores 41 in (b1).
- The opinions in (a2) take longer to converge than (a1), and (b2) take longer to converge than (b1). Individuals with initial extreme opinions have lower affinity scores with others. The presence of these individuals slows down the group consensus process.
- The coevolution of affinity and opinion accelerates each other, with (a3) and (b3) converges faster than (a2) and (b2) respectively.
- When affinity changes with opinion, individuals with low initial affinity converge slowly, but their convergence speeds up as affinity increases with decreasing opinion differences. As shown in (b3), individuals with extreme initial opinion scores and thus low initial affinity scores have slower opinion evolution at the beginning and become faster later.

**Fig 2:**
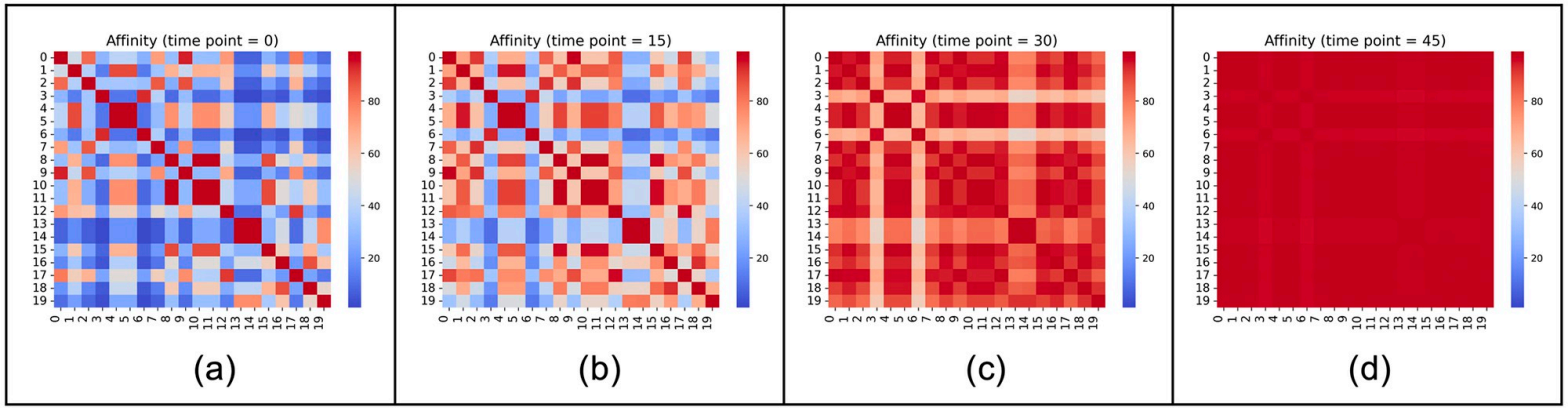
Affinity score matrix at four time points (*μ* = 0.04) (a) n = 0, (b) n = 15, (c) n = 30, (d) n = 45. Blue color represents lower affinity and red color higher affinity.

Fig 2 illustrates the evolution of affinity during the interactive process at four selected time points (*μ* = 0.04). At n = 0, the affinity matrix is predominantly blue, indicating a low initial affinity level. As interaction progresses, more red blocks appear, signifying the increase in affinity. The affinity score approaches its highest value eventually when the opinions converge.

## Experimental data description

### Experiment description

In this work, we utilize an opinion and affinity (attraction) dataset from a previous study [29] to test our proposed three models. We provide a brief overview of the experiments and the data collection process below. For a more detailed description of the experiment setup and the dataset, interested readers are encouraged to refer to the original article.

The participants in the study were recruited from the University of Groningen, and the experiments were conducted using an anonymous communication system. There were 100 participants, and a total of 850 valid recorded interactions/pairs between two individuals were collected (380 pairs in the control group and 470 pairs in the manipulated group).

A total of 19 topics were used in the experiment, and some sample topics are listed in Table 1. For each specific topic, participants were asked to record their opinion score on a scale from 0 to 100, indicating different stances towards the given topic. Additionally, participants were requested to record the affinity score indicating the degree of liking towards their partner. The affinity score was measured on a scale of 0 to 100, with 0 indicating very strong disliking and 100 representing the highest level of liking.

**Table 1:** Examples of topics.

Sample topics
1. The government should subsidize public transport in 0 … 100 percent.
2. A demonstration needs police protection. Organizers should pay 0 … 100 percent of the costs of this.
3. 0 … 100 percent of immigrants who come to the Netherlands for economic reason should receive a residence permit.
4. …

To demonstrate the potential effects of initial affinity on later opinion shifts, a subset of interactions was randomly selected. In these interactions, the initial affinity of individuals towards their partners (sources) was manipulated beforehand with the aim of inducing disliking. In this study, we refer to the interactions with and without such manipulations as the control group and the manipulated group, respectively. The mean initial affinity score of the control group was *μ* = 60.22 with a standard deviation of *σ* = 20.88, which is higher than that of the manipulated group with a mean of *μ* = 56.57 and a standard deviation of *σ* = 21.85.

### Dataset description

**Fig 3:**
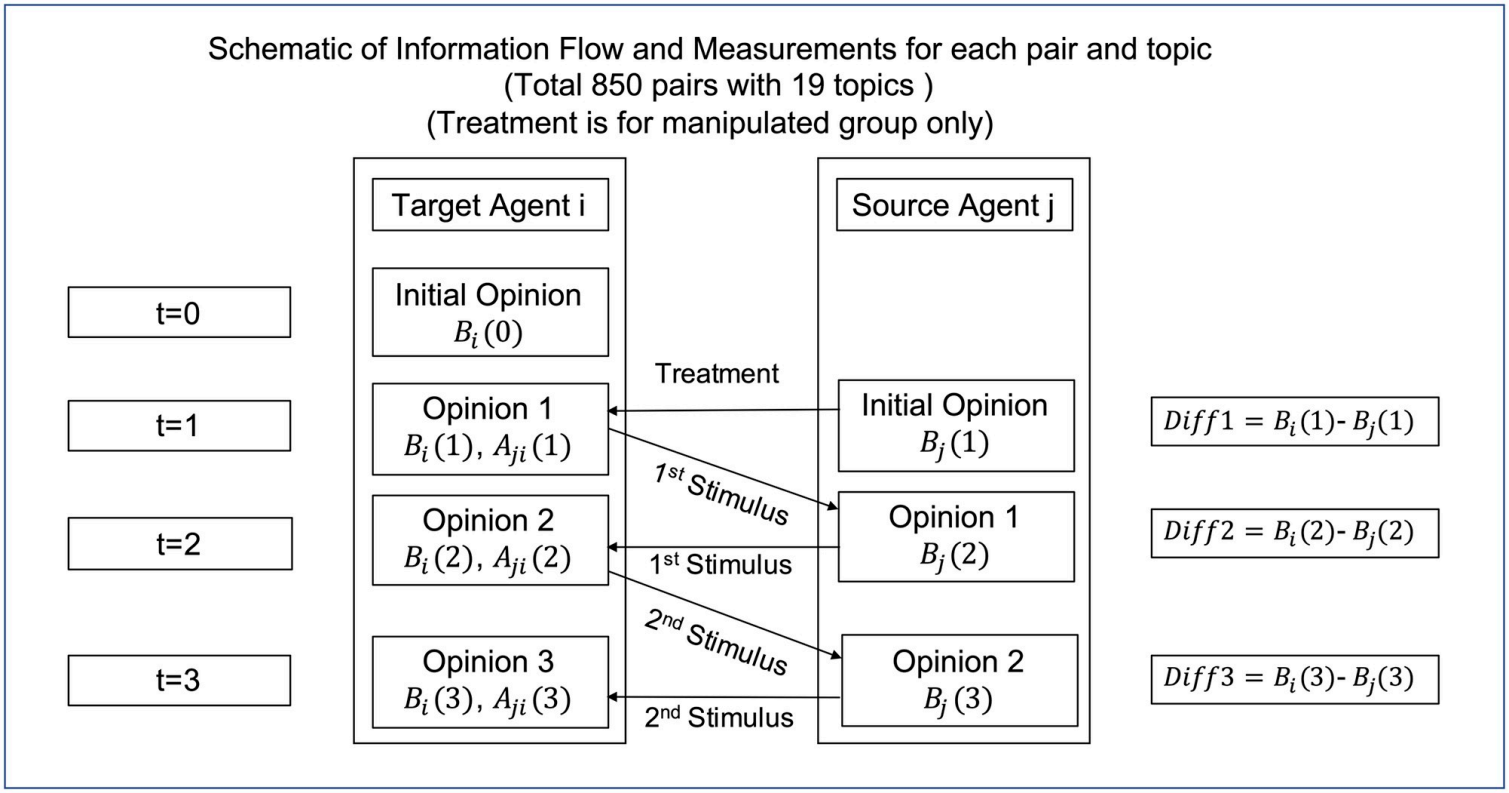
Schematic of interaction process.

Each target individual was randomly paired with a source individual, and their opinions on a selected topic were recorded. Fig 3 illustrates the interaction process. Initially, the opinions of the individuals were separately measured (*B*_*i*_(0) for the target and *B*_*j*_(1) for the source), reflecting their raw opinions before any communication took place.

For the control group, the source’s initial opinion was directly displayed to the target. However, for the manipulated group, the source’s initial opinion was displayed to the target after performing a disliking manipulation.

The manipulation was implemented with a combination of three methods to keep a variation in lowering affinity rating from partner individual to target individual, including selecting of a student partner with areas of study different from the target, playing a monetary win-or-loss confronting game between the two individuals, and sending a stigmatizing message from the partner to the target. More details about the manipulation can be found in [29].

After receiving the source’s initial opinion, the updated opinion score of the target and their evaluation of the corresponding source’s affinity score were recorded (*B*_*i*_(1), *A*_*ji*_(1)). Then, the target’s opinion score was displayed to the source, and the source’s opinion score was displayed to the target (the first stimulus), marking the completion of the first round. During the interactive process, the updated opinions of both targets and sources, as well as the affinity score of sources evaluated by the targets, were recorded (*B*_*i*_(2), *A*_*ji*_(2), *B*_*j*_(2)). This process was repeated, and the second round began. The target’s updated opinion score was displayed to the source, and the source’s updated opinion score was displayed to the target (the second stimulus), followed by the recording of further updated opinion scores and affinity scores (*B*_*i*_(3), *A*_*ji*_(3), *B*_*j*_(3)). In this experiment, only the affinity score evaluated by the target was recorded, while the affinity score evaluated by the source was not recorded. Table 2 provides descriptions for the notations used.

**Table 2:** Notations.

Notation	Description
*B*_*i*_(0)	Target’s opinion at the very beginning.
*B*_*j*_(1)	Source’s opinion at the very beginning.
*B*_*i*_(1)	Target’s updated opinion after the initial manipulation.
*B*_*j*_(2)	Source’s opinion after the first stimulus.
*B*_*i*_(2)	Target’s opinion after the first stimulus.
*B*_*j*_(3)	Source’s opinion after the second stimulus.
*B*_*i*_(3)	Target’s opinion after the second stimulus.
*A*_*ji*_(1)	Target’s affinity evaluation towards the source after the initial manipulation.
*A*_*ji*_(2)	Target’s affinity evaluation towards the source after the first stimulus.
*A*_*ji*_(3)	Target’s affinity evaluation towards the source after the second stimulus.

It should be noted that the pairs of source and target for these multiple interactions were not fixed, and the assigned topics for each pair were also not fixed. The measurements of affinity scores may be influenced by subjective evaluation reference points that vary among individuals. Some targets may consistently give higher or lower scores to their partners, resulting in different affinity scores for the same source given by different targets. Similarly, the measurements of opinion scores are also individual-dependent, as they are generated based on individuals’ subjective evaluation reference points, even when a measurement standard is provided.

Therefore, the dataset contains a considerable amount of noise, and the opinion difference between source *j* and target *i* after the *n*-*th* round of interaction, Δ*B*_*ji*_(*n*) = *B*_*j*_(*n*) − *B*_*i*_(*n*), may be influenced by the subjective evaluation references of the two individuals. However, the opinion change of target *i* during the *n*-*th* round, *B*_*i*_(*n* + 1) − *B*_*i*_(*n*), is much less affected by the noise, as an individual’s reference point does not change significantly within a short period of time for the same topic. Therefore, the difference of Δ*B*_*ji*_(*n*) is largely unaffected by such biases, as shown in Eq 10.

$$\begin{array}{c} \begin{array}{c} \Delta B_{ji}\left(n+1\right)-\Delta B_{ji}\left(n\right)=\left(B_{j}\left(n+1\right)-B_{i}\left(n+1\right)\right)-\left(B_{j}\left(n\right)-B_{i}\left(n\right)\right)\\ =\left(B_{j}\left(n+1\right)-B_{j}\left(n\right)\right)-\left(B_{i}\left(n+1\right)-B_{i}\left(n\right)\right) \end{array} \end{array}$$ (10)

So, the left-hand side of Eq 8, which appears in Model 3, is much less affected by subjective references. The difference |Δ*B*_*ji*_(*n* + 1)| − |Δ*B*_*ji*_(*n*)|, which appears in Eq 3, Model 1, is less affected when Δ*B*_*ji*_(*n* + 1) and Δ*B*_*ji*_(*n*) have the same sign. The subjective reference effect may be significant if the two terms have opposite signs, but such cases rarely occur within a short time interval.

## Statistical model tests

### Test for Model 1

**Fig 4:**
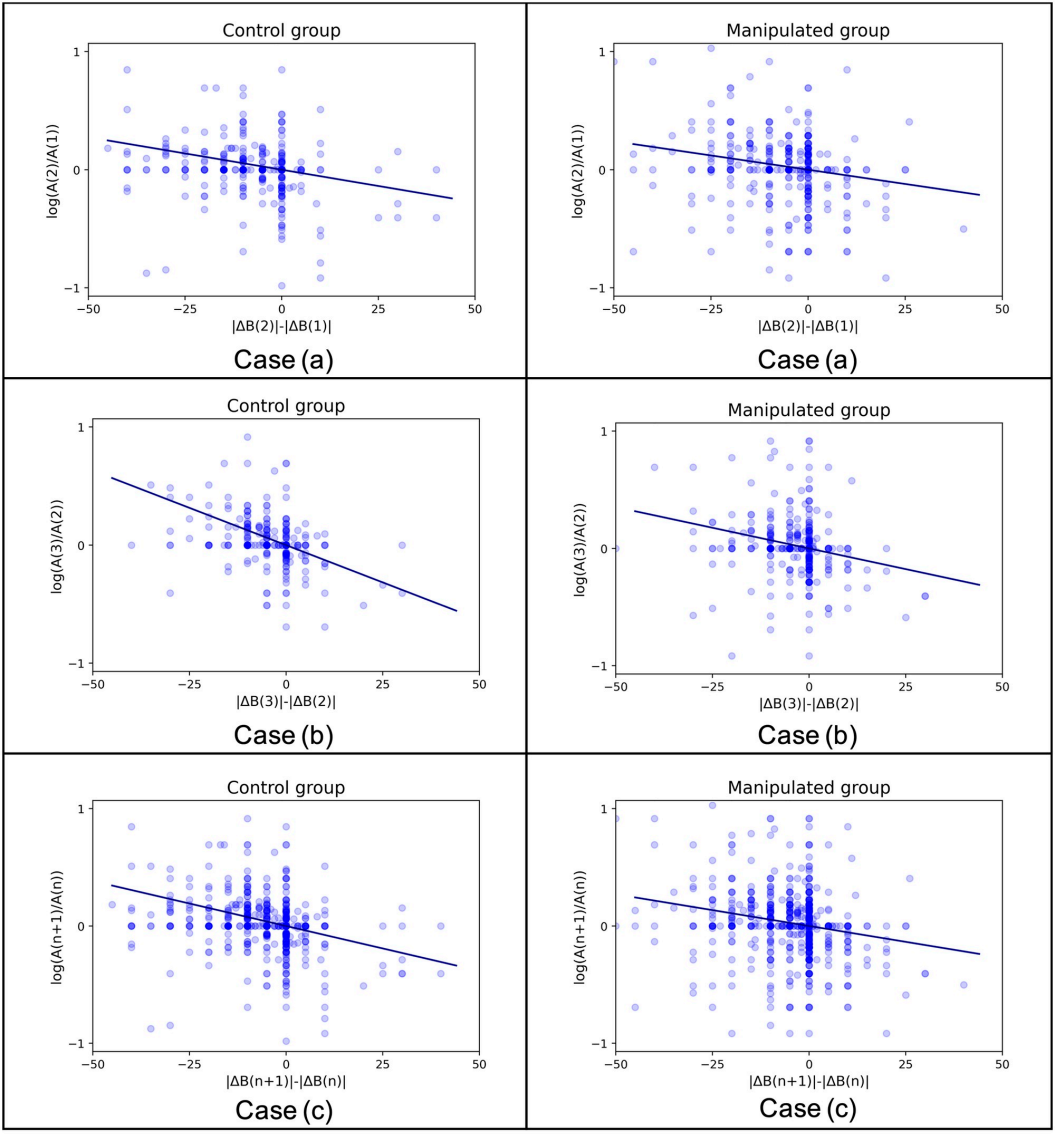
Model 1(with zoom-in). Case (a), (b), and (c) represent the three previously mentioned cases. The horizontal axis represents the change in absolute opinion difference between the pair, while the vertical axis represents the logarithm of affinity change. The scatter points depict the relationships between these two changes after the interactions, with darker colors indicating overlapping points. The deep blue fitted lines are generated using the OLS method. For illustrative purposes, we have zoomed in on the central part of the complete figures. The complete figures can be found in S1 Appendix in S1 File.

In this subsection, we test Model 1, the value homophily model, as shown by Eq 3. To ensure meaningful logarithmic calculations, we exclude interactions with extreme affinity scores of either 0 or 100, resulting in 359 interactions for the control group and 443 interactions for the manipulated group. Fig 4 illustrates the relationship between the term $ln[\frac{A_{ji}\left(n+1\right)}{A_{ji}\left(n\right)}]$ and [|Δ*B*_*ji*_(*n* + 1)| − |Δ*B*_*ji*_(*n*)|] for both the control group and the manipulated group. Each group is studied in three cases: (a) data from the first stimulus, (b) data from the second stimulus, and (c) the combination of (a) and (b).

It can be found from Fig 4 that a large portion of points are distributed in the second and the third quadrant, which shows that “positive shift” of opinion difference is much more common in the experiment.

Specifically, in Fig 4-Case (c), for the control group, there are 356 points distributed in the second and third quadrants, compared to only 70 points in the first and fourth quadrants; for the manipulated group, there are 390 points in the second and fourth quadrants, compared to only 96 points in the first and fourth quadrants. Among these points, more points lie in the second quadrant than in the third one, indicating that pairs with decreasing opinion difference tend to increase the affinity level. On the other hand, among those “negative shift” points, which lie in the first and the fourth quadrants, most of them lie in the fourth quadrant and only a few in the first quadrant, which indicates that pairs with increasing opinion difference tend to decrease the affinity level. These observations imply a negative relationship between opinion difference shift and affinity shift.

We employed the well-known ordinary least squares (OLS) method [32] to validate the linear relationship between the explanatory variable [|Δ*B*_*ji*_(*n* + 1)| − |Δ*B*_*ji*_(*n*)|] and the response variable $ln[\frac{A_{ji}\left(n+1\right)}{A_{ji}\left(n\right)}]$. The fitted lines for all three cases in both groups, as depicted in Fig 4, indicate the existence of linear relationships between the two variables, despite the noisy nature of the data distribution.

Table 3 below shows the coefficient estimation in Model 1 for all six cases, including the estimation of coefficient −*μ*, the standard error of the estimation, the 95% confidence intervals of the coefficient estimation, and the corresponding p-value.

**Table 3:** Statistical test results of Model 1.

Group	Case	Coefficient	Standard Error	95% CI Left	95% CI Right	p-value
Control	Case(a)	-5.9E-03	1.4E-03	-8.2E-03	-2.8E-03	<0.001
Control	Case(b)	-1.3E-02	1.4E-03	-1.5E-02	-9.9E-03	<0.001
Control	Case(c)	-7.7E-03	9.8E-04	-9.6E-03	-5.7E-03	<0.001
Manipulated	Case(a)	-4.8E-03	1.2E-03	-7.2E-03	-2.4E-03	<0.001
Manipulated	Case(b)	-7.1E-03	1.8E-03	-1.1E-02	-3.5E-03	<0.001
Manipulated	Case(c)	-5.4E-03	9.8E-04	-7.3E-03	-3.5E-03	<0.001

As shown in Table 3, for all three cases of both groups, the coefficient estimates of the explanatory variable [|Δ*B*_*ji*_(*n* + 1)| − |Δ*B*_*ji*_(*n*)|] are significant at the p <0.001 level. It can also be noticed that, for all cases, the estimates of coefficients are all negative, and their corresponding 95% confidence intervals all lie in the negative range as well. This implies that smaller opinion differences usually come with higher affinity score, and vice versa. The results validate the Model 1 and match the viewpoints from previous studies [4, 33, 34].

For both the control group and the manipulated group, Case (b) exhibits larger absolute coefficient values compared to Case (a). This suggests that the value homophily effects become stronger at a later stage during brief interactions. Additionally, as shown in Fig 4, the data points in Case (a) appear more scattered than those in Case (b). This observation aligns with the findings of Gravetter and Forzano [35], who suggest that the presence of a novelty effect may introduce more noise in data collected at the beginning of an experiment, possibly due to factors such as participants’ mental state or unfamiliarity. Individuals tend to be relatively insensitive to early opinion differences and change their affinity slowly. However, as the interactive communications progress, the changes in affinity scores become more pronounced. Therefore, the parameter estimated in Case (b) provides a more reliable estimation that can be used for later rounds of interactions.

For both Case (a) and Case (b), the absolute coefficient values of the manipulated group are smaller than those of the control group, indicating that the affinity scores change less for the manipulated group compared to the control group. This can be attributed to the fact that individuals in the manipulated group experienced a negative first impression, while individuals in the control group did not. This observation is consistent with the findings of Gilron and Gutchess [36] and Baxter et al. [37], which suggest that first impressions tend to have a lasting impact and influence individuals’ subsequent thoughts and behaviors.

### Test for Model 2

**Fig 5:**
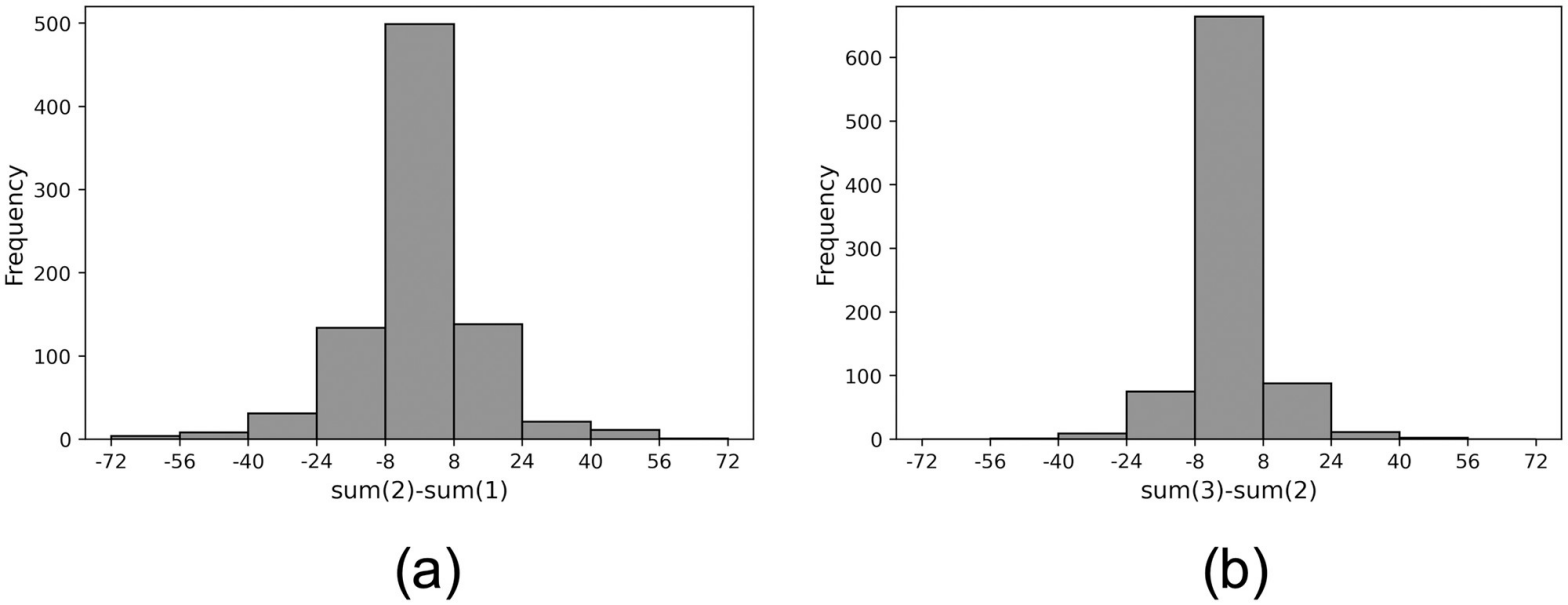
Frequency distribution of differences between the summations of opinion scores at two different time points. (a) Between sum(2) and sum(1), (b) Between sum(3) and sum(2).

In this subsection, we test Model 2: the sum of opinions of the source and target before and after communication being equal. We denote the sum of opinion scores after the *n*-*th* round as *sum*(*n*) = *B*_*i*_(*n*) + *B*_*j*_(*n*). Fig 5(a) and 5(b) show the frequency of occurrence for *sum*(2) − *sum*(1) and *sum*(3) − *sum*(2), respectively. The figures indicate that the difference in the summation between two consecutive time points is mainly within a narrow range (-8, 8), compared to the largest possible range (-200, 200).

**Fig 6:**
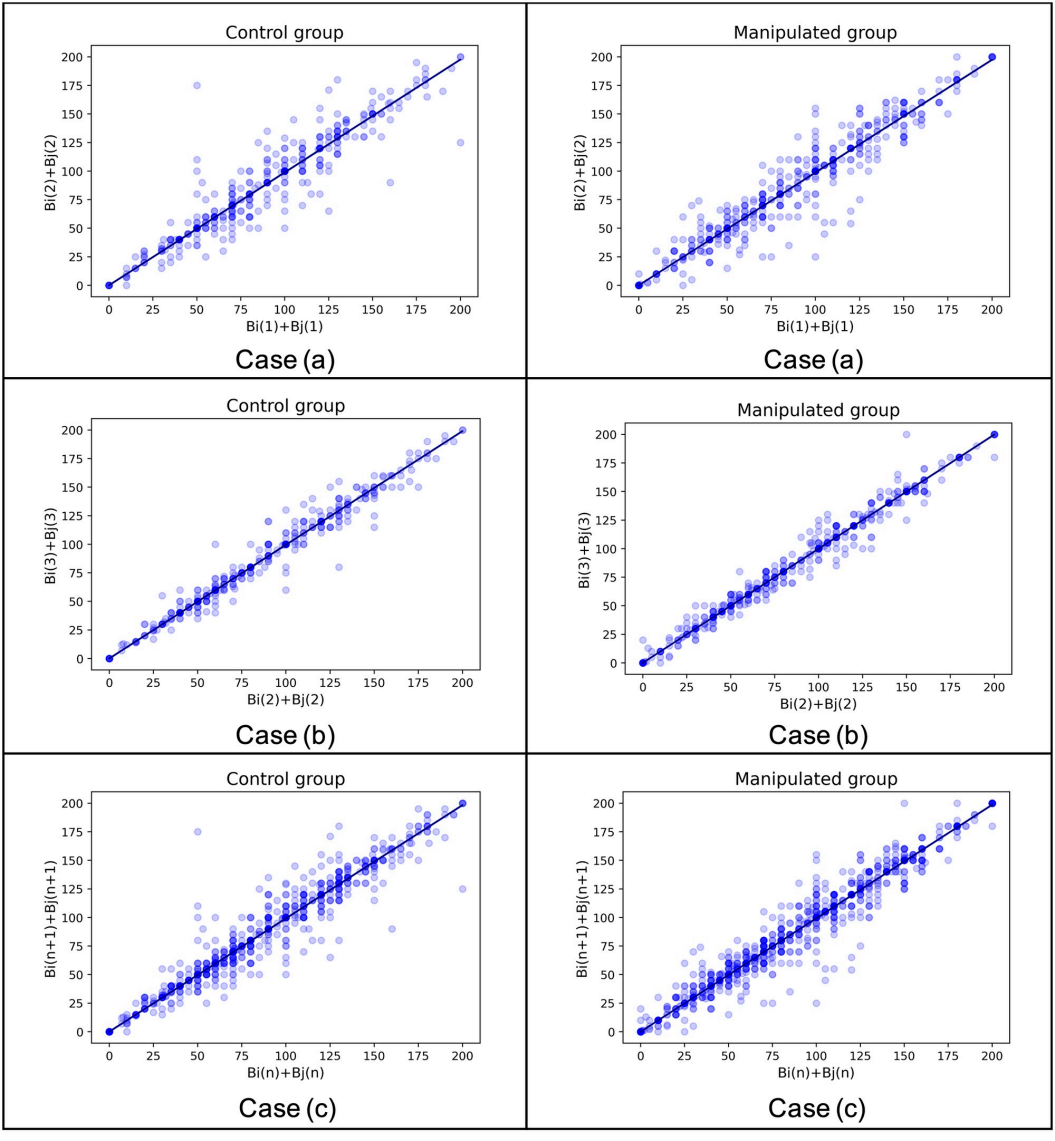
Model 2. Case (a), (b), and (c) represent the three aforementioned cases. The horizontal axis represents the opinion summation at the current time point, while the vertical axis represents the opinion summation at the next time point. The scatter points depict the distribution of these two sums, with darker colors indicating overlapping points. The deep blue fitted lines are generated using the OLS method.

Fig 6 presents all three cases for both groups, depicting the distribution of opinion summation at two different time points. The three cases for each group include: (a) data from the first stimulus, (b) data from the second stimulus, and (c) the combination of (a) and (b). The scatter points exhibit a dense distribution around the fitted line. Table 4 displays the estimation results of Model 2. The relationship between sum(n+1) and sum(n) is well approximated by the linear model, with a slope that is very close to one.

**Table 4:** Statistical test results of Model 2.

Group	Case	Coefficient	Standard Error	95% CI Left	95% CI Right	p-value	p-value (equi-test)
Control	Case(a)	9.9E-01	8.0E-03	9.7E-01	1.0E+00	<0.001	<0.001
Control	Case(b)	1.0E+00	4.0E-03	9.9E-01	1.0E+00	<0.001	<0.001
Control	Case(c)	9.9E-01	5.0E-03	9.8E-01	1.0E+00	<0.001	<0.001
Manipulated	Case(a)	9.9E-01	7.0E-03	9.7E-01	1.0E+00	<0.001	<0.001
Manipulated	Case(b)	1.0E+00	3.0E-03	9.9E-01	1.0E+00	<0.001	<0.001
Manipulated	Case(c)	9.9E-01	4.0E-03	9.9E-01	1.0E+00	<0.001	<0.001

In addition, we performed an equivalence test [38] for all cases using an equivalence interval of (-3, +3). The null hypothesis (H_0_) states that the difference between sum(n) and sum(n+1) falls outside the interval (-3, +3), while the alternative hypothesis (H_1_) states that the difference falls inside the interval (-3, +3).

The results of the equivalence test are presented in the last column of Table 4. It can be observed that for all cases, the p-values are less than 0.001, indicating that the null hypothesis can be rejected at the 0.001 *α* level. Therefore, it can be concluded that the opinion summations before and after the interaction can be considered statistically equivalent.

### Test for Model 3

**Fig 7:**
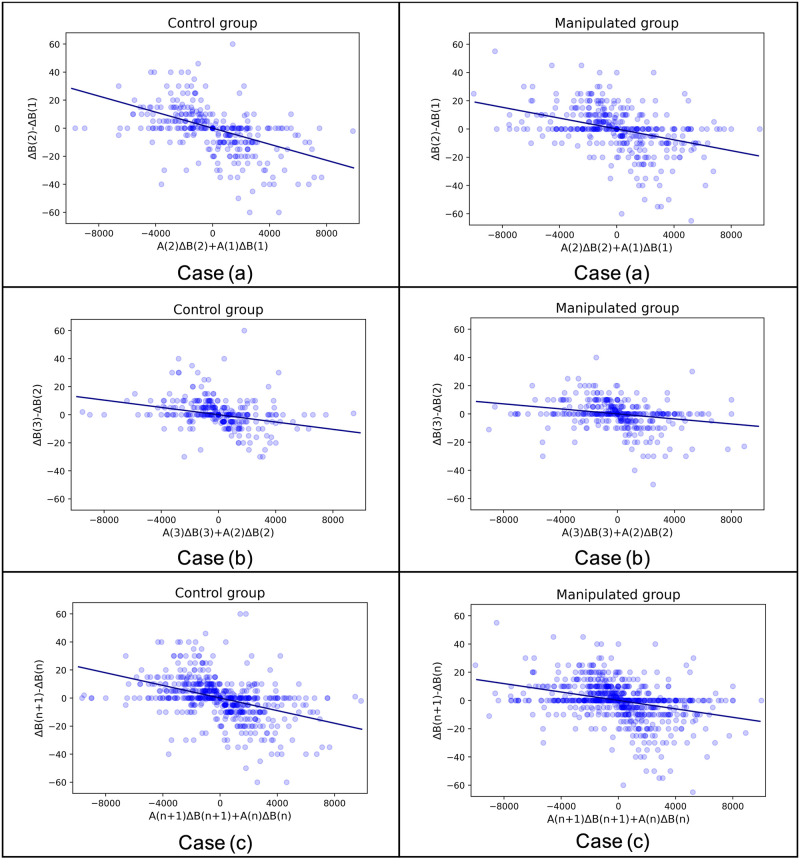
Model 3. Case (a), (b), and (c) represent the three aforementioned cases. The horizontal axis represents the constructed variable, while the vertical axis represents the temporal change of the opinion score difference. The scatter points illustrate the distribution of these two variables, with darker colors indicating overlapping points. The deep blue fitted lines are drawn using the OLS method.

In this subsection, we examine Model 3, which focuses on the temporal evolution of the opinion difference between two interactive individuals, as depicted by Eq 8. Fig 7 showcases the relationship between the term [Δ*B*_*ji*_(*n* + 1) − Δ*B*_*ji*_(*n*)] and [*A*_*ij*_(*n* + 1)Δ*B*_*ji*_(*n* + 1) + *A*_*ij*_(*n*)Δ*B*_*ji*_(*n*)] for both the control group and the manipulated group. The figure includes three cases for each group.

Table 5 presents the coefficient estimation in Model 3 for all cases, including the estimation of the coefficient −*α*, the standard error of the estimation, the 95% confidence interval, and the corresponding p-value. For all three cases in both groups, there is a significant negative relationship between [Δ*B*_*ji*_(*n* + 1) − Δ*B*_*ji*_(*n*)] and [*A*_*ij*_(*n*+ 1)Δ*B*_*ji*_(*n* + 1) + *A*_*ij*_(*n*)Δ*B*_*ji*_(*n*)], with statistically significant negative coefficient estimates (p <0.001).

**Table 5:** Statistical test results of Model 3.

Group	Case	Coefficient	Standard Error	95% CI Left	95% CI Right	p-value
Control	Case(a)	-2.9E-03	2.9E-04	-3.4E-03	-2.3E-03	<0.001
Control	Case(b)	-1.3E-03	2.2E-04	-1.7E-03	-8.6E-04	<0.001
Control	Case(c)	-2.2E-03	1.9E-04	-2.6E-03	-1.9E-03	<0.001
Manipulated	Case(a)	-1.9E-03	2.3E-04	-2.4E-03	-1.5E-03	<0.001
Manipulated	Case(b)	-8.9E-04	1.7E-04	-1.2E-03	-5.6E-04	<0.001
Manipulated	Case(c)	-1.5E-03	1.5E-04	-1.8E-03	-1.2E-03	<0.001

For both the control group and the manipulated group, Case (b) has smaller absolute coefficient values compared to Case (a). This suggests that the influence of communication in changing individuals’ opinion differences may diminish over time. In the early stages of communication, people with different opinions converge more quickly. However, as the rounds of communication progress, this impact weakens. Individuals tend to hold more firmly rooted opinions and exhibit less change in later stages. However, it is important to note that the data points in Case (a) are more scattered than in Case (b), indicating the need for further experimental results with additional rounds of interactions to draw more robust conclusions.

For both Case (a) and Case (b), the absolute coefficient values of the manipulated group are smaller than those of the control group. Additionally, there is a significant coefficient difference between the control and manipulated groups in Case (a). This indicates the influence of affinity on opinion change. According to the study by Cialdini [39], mutual affinity increases the likelihood of an individual being influenced by another individual. Individuals in the manipulated group hold a poorer impression of their partners and, as a result, are more reluctant to change their opinions.

### Comparison with the DeGroot model

The idea of linear assimilative social influence has been used in many previous works, and the DeGroot Model is one of the representatives. In DeGroot’s model, individual *i* updates his opinion through a weighted sum of his own opinion and the opinion from individual *j* in pairwise communication. Notably, in DeGroot’s model, these weights, which represent the influence of one person to another person, are fixed and independent of their opinion difference. This constitutes the most significant difference between DeGroot’s model and ours, as we believe that such weights should be influenced by the varying affinity.

With a straightforward mathematical transformation, DeGroot’s model can be expressed by Eq 11, *γ* is a constant parameter and represents the linear assimilative influence. We conducted tests comparing the performance of this model with that of our Model 3, and the main results are presented in Table 6.

$$\begin{array}{c} \begin{array}{c} \Delta B_{ji}\left(n+1\right)-\Delta B_{ji}\left(n\right)=\gamma \left(\Delta B_{ji}\left(n+1\right)-\Delta B_{ji}\left(n\right)\right) \end{array} \end{array}$$ (11)

**Table 6:** Performance comparison between Model 3 and the DeGroot model.

		Model 3			DeGroot Model		
Group	Case	Coefficient	p-value	Adjusted R⌃2	Coefficient	p-value	Adjusted R⌃2
Control	Case(a)	-2.9E-03	<0.001	0.215	-1.4E-01	<0.001	0.192
Control	Case(b)	-1.3E-03	<0.001	0.083	-6.4E-02	<0.001	0.081
Control	Case(c)	-2.2E-03	<0.001	0.164	-1.1E-01	<0.001	0.147
Manipulated	Case(a)	-1.9E-03	<0.001	0.132	-1.0E-01	<0.001	0.144
Manipulated	Case(b)	-8.9E-04	<0.001	0.059	-5.1E-02	<0.001	0.077
Manipulated	Case(c)	-1.5E-03	<0.001	0.102	-8.1E-02	<0.001	0.115

We tested the DeGroot model on both of the control and the manipulated group across all three cases. It is shown that the estimated coefficient has strong statistical significance in all cases with p-values <0.001. Taking a step further, we compared the adjusted R-squared values between the DeGroot model and ours. Higher adjusted R-squared values indicate better fitting performance.

As shown in the Table 6, in the control group, our model consistently outperforms the DeGroot model, whereas in the manipulated group, the DeGroot model exhibits better performance. In situations with a friendly affinity level, affinity facilitates the assimilation process, resulting in higher opinion convergence efficiency. This suggests that when there is a stronger affinity with the other party, opinions are updated more rapidly. However, when affinity is manipulated to a low level, the affinity effect becomes negligible, resulting in a much slower opinion convergence.

In summary, we believe that the introduction of the concept “affinity” is important. In the control cases, Model 3 performs better than the classical model consistently. In the manipulated cases, Model 3 does not perform as well as the DeGroot model. The convincing reasons for this inferior performance await further experimental and analytical research.

## Discussion

Valuable insights have been gained from the proposed models and experimental results.

- Model 1 examines the negative relationship between homophily affinity and opinion difference between the individuals. In the realm of politics, conflicts over specific issues or policies can strain relationships. For instance, the widening divergence of opinions on economic, political, and other topics between the UK and other European Union (EU) countries created significant divisions, ultimately culminating in Brexit.
- The concept underlying Model 2 is: when pair of individuals engage in a conversation, they tend to update their opinions towards a middle ground or away from each other. The sum of the opinion scores does not change. For instance, it is uncommon for two individuals who both think global warming a big issue, albeit to varying degrees, to consider global warming no longer important after communication.
- Model 3 explores the influence of affinity in the opinion assimilation process. Based on the experimental results, assimilation tends to occur more rapidly in situations with higher affinity levels. It is often observed that countries with amicable diplomatic relationships are more inclined to collaborate in formulating strategies and establishing common goals. For example, the strong relationship between the UK and the USA has facilitated their alignment on various global challenges, such as human rights and counterterrorism.

Our model distinguishes itself from traditional models by introducing a novel concept called “affinity,” which plays an important role in influencing the updates of individuals’ opinions. In classic models, the influence weight of one individual’s opinion on another individual is typically considered fixed. However, Model 3 assumes that the affinity plays a role in the update rate of opinion change. This assumption has been supported by the statistical test results presented in Table 6.

Besides, observations from Fig 4 indicate that the affinity changes during communication and cannot be treated as a constant. It can be noticed from the experimental results of Model 1 that, increased affinity is accompanied by decreased opinion difference. And the affinity of people in the manipulated group, which are ones with a worse initial impression towards their partners, are more insensitive to changes in opinion difference. This low affinity contributes to reduced communication efficiency, as shown by the smaller absolute values of estimated coefficients in the manipulated group in Model 3.

This prompts us to consider that low affinity weakens the effectiveness of communication, making persuasion more difficult to achieve. Such dislike may be accompanied by a large opinion difference. Since the 1970s, the voting divergence between the two parties in the US Congress has increased and persisted. Low communication efficiency, which arises from a low affinity level, may contribute to such persistent divergence. And this highlights the importance of taking measures to prevent expanding opinion divergence from transforming into entrenched antipathy towards opposing parties, concerning the negative relationship between opinion difference change and affinity change pointed by our Model 1.

## Conclusion

Over the years, the analytical form of the value homophily relationship between affinity and opinion difference has not been developed, and the inclusion of affinity in the opinion dynamics model has also not been explicitly expressed, even though many of these models have incorporated the effect of value homophily in various ways. The evolution of affinity and opinion is rather complex, and accurately characterizing the underlying micro-relations is essential for gaining insights into the affinity-opinion system and its properties.

This paper proposes two analytical relationships and tests them using empirical experimental dataset. The first relationship is for value homophily, as expressed in Eq 1, which captures the relationship between affinity score and opinion score difference between two individuals. The second relationship involves the inclusion of affinity in the opinion dynamics model, as shown in Eq 4. Due to the inherent noise in the dataset, direct validation of these two relationships can be challenging. We derive three micro-mechanisms (Eqs 3, 6 and 8) based on these two analytical relationships and test them using experimental dataset from Takács et al. [29]. These three test models are the value homophily model, the temporal evolution of the summation of the opinion scores, and the evolution of the difference of opinion scores, which show how affinity and opinion evolve and affect each other. Promising statistical test results have been obtained for all three models, thereby validating the value homophily relationship and its inclusion in the opinion dynamics. These two micro mechanisms can be further applied to the modeling of macro-scale opinion dynamics that involve a larger number of individuals.

It should be noted that the dataset used in this study is noisy, although the two relationships have been reasonably well validated. This does not rule out the validity of other closely related analytical forms, which are discussed in detail in S2 Appendix in S1 File. Further research is necessary to gain a better understanding and more conclusive quantification of the relationship between affinity and opinion shift, and its integration into opinion dynamics models. This includes the following considerations:

- Conducting better-designed and controlled experiments involving a larger number of individuals and multiple rounds of interactions. It is anticipated that the value homophily coefficient *μ* in Eq 1 is correlated with societal characteristics such as race, ethnicity, sex, age, etc. Quantifying this coefficient for different groups of individuals is essential.
- To apply and generalize the two-individual relationships to model macro-scale opinion dynamics, experiments need to be conducted to test the relationships using affinity score and opinion score measurements from a larger population of interacting individuals.
- Applying the developed two-individual relationships to macro-scale problems in order to investigate the evolution of opinion dynamics. This can include studying interactions within different social networks, interactions among multiple groups of individuals, interactions involving multiple related opinion topics, and the evolution of affinity among individuals due to these interactions.

Addressing these aspects will contribute to a deeper understanding of opinion dynamics and the role of affinity in shaping collective opinions on a larger scale.

## Supporting information

S1 File (PDF)
