# Supplementary material for: Affinity, value homophily, and opinion dynamics: The coevolution between affinity and opinion
Source: PLoS One. 2023 Nov 27;18(11):e0294757. doi: 10.1371/journal.pone.0294757 (PMC10681268; doi:10.1371/journal.pone.0294757)
Supplement: S1 File — (PDF) [file pone.0294757.s001.pdf]

S1 Appendix. Complete figures of Model 1. Full scale figures for Model 1 are provided here as Fig 8 for the completeness.

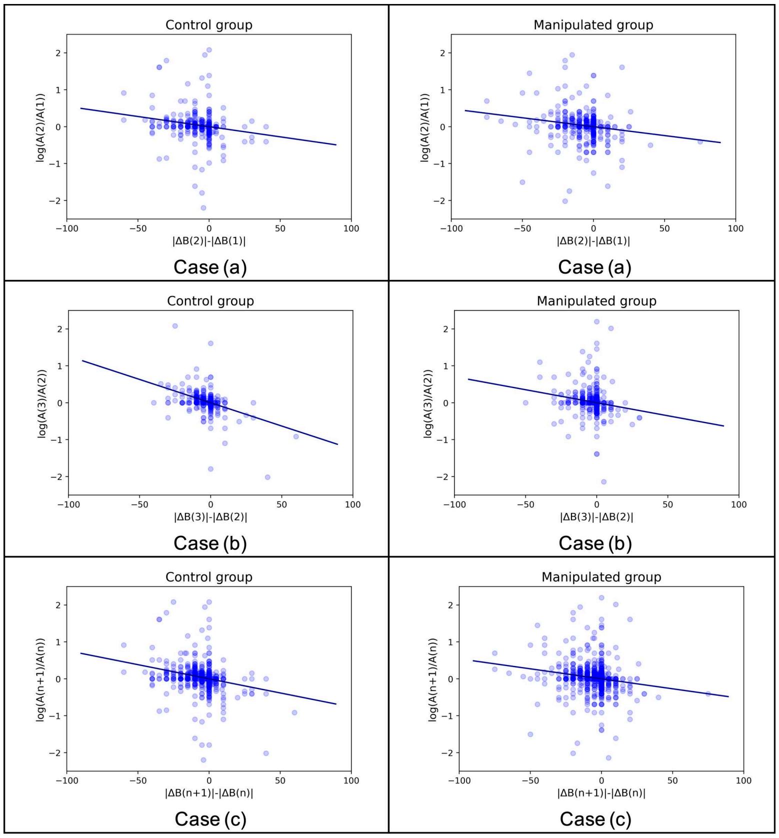

Fig 8. Model 1 without zoom in. Case (a), (b), and (c) refer to the aforementioned three cases. The horizontal axis represents the change in absolute opinion difference, while the vertical axis represents the logarithm of affinity change. The scatter points represent the corresponding changes in these two variables during the interactions, with darker colors indicating overlapping points. The deep blue fitted lines are drawn using the OLS method.

S2 Appendix. A variant of value homophily relationship.

The value homophily relationship shown by Eq 1 may also be expressed as,

$$A_{ji}(n) = A_{ji}(n_{\Delta B_{ji}=0})e^{-\mu \Delta B_{ji}{}^2(n)} = A_{ji}(n_{\Delta B_{ji}=0})e^{-\mu [B_j(n)-B_i(n)]^2} \tag{12}$$

Model B1 can be obtained from Eq 12, as shown by Eq 13.

$$ln[\frac{A_{ij}(n+1))}{(A_{ij}(n)}] = -\mu[\Delta B_{ij}^2(n+1) - \Delta B_{ij}^2(n)] \tag{13}$$

Table 7 presents the statistical test results of Model B1. When comparing these results with those of Model 1, we observe a slightly weaker significance in this variant model, as indicated by the p-values in case (a) for both the control group and the manipulated group.

Fig 9 and Fig 10 depict the distribution of the two constructed variables, with Fig 9 providing a zoomed-in version. It can be observed that the majority of the points lie in the second and fourth quadrants, indicating a negative relationship between the shift in opinion difference and the change in affinity.

Table 7. Statistical test results of the variant Model B1.

Group	Case	Coefficient	Standard Error	95% CI Left	95% CI Right	p-value
Control	Case(a)	-6.5E-05	2.1E-05	-1.1E-04	-2.4E-05	<0.01
Control	Case(b)	-1.5E-04	2.3E-05	-2.0E-04	-1.1E-04	<0.001
Control	Case(c)	-9.0E-05	1.5E-05	-1.2E-04	-6.0E-05	<0.001
Manipulated	Case(a)	-4.9E-05	1.5E-05	-7.9E-05	-1.9E-05	<0.01
Manipulated	Case(b)	-9.5E-05	2.6E-05	-1.5E-04	-4.3E-05	<0.001
Manipulated	Case(c)	-5.9E-05	1.3E-05	-8.4E-05	-3.3E-05	<0.001

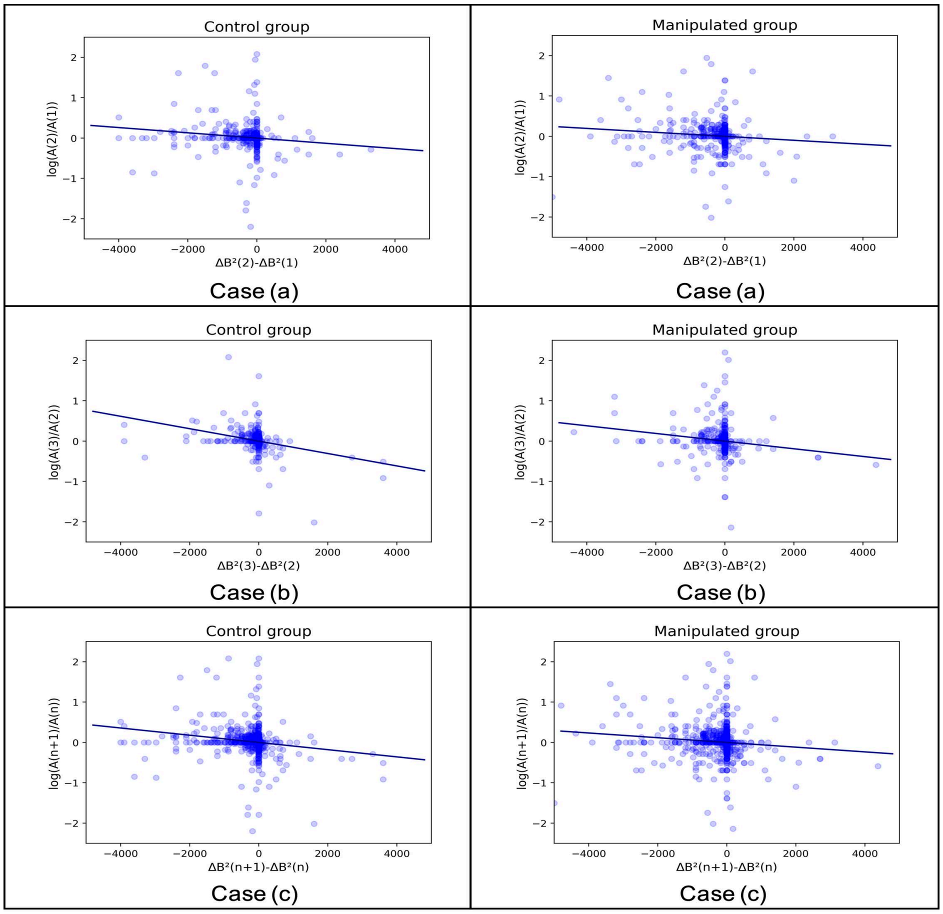

Fig 9. Model B1 with zoom-in. Case (a), (b), and (c) denote the aforementioned three cases. The horizontal axis represents the change between the square of opinion difference, while the vertical axis represents the logarithm of affinity change. The scatter points depict the corresponding changes of these two variables due to the interactions, with darker colors indicating overlapping points. The deep blue fitted lines are drawn using the OLS method.

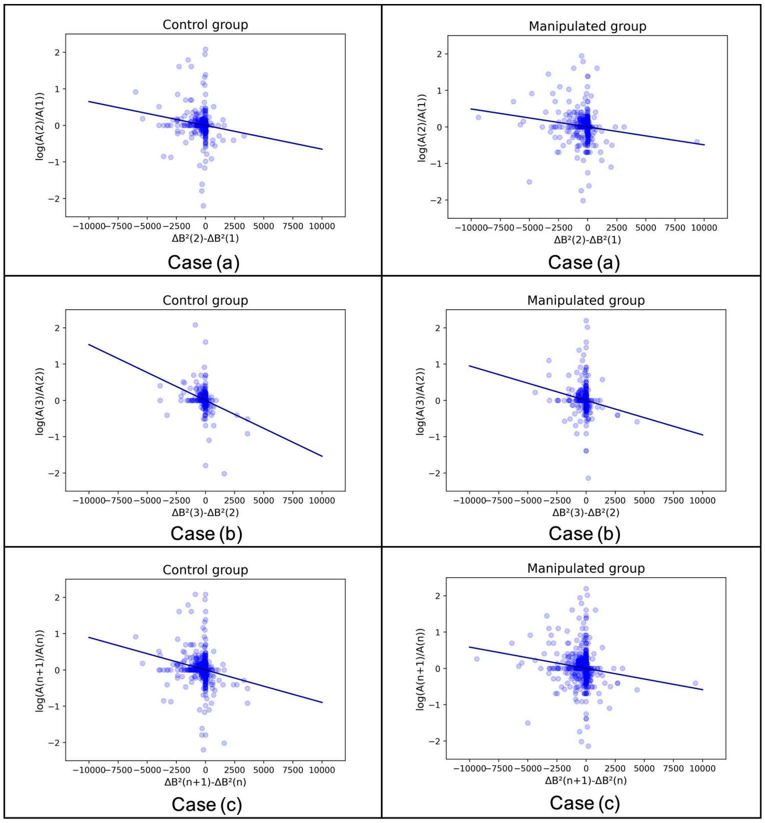

Fig 10. Model B1 without zoom-in. Case(a), (b), and (c) denote the aforementioned three cases.
